# Lack of inducible nitric oxide synthases attenuates leukocyte–endothelial cell interactions in retinal microcirculation

**DOI:** 10.1136/bjo.2007.131151

**Published:** 2008-04-25

**Authors:** D Iwama, S Miyahara, H Tamura, K Miyamoto, F Hirose, N Yoshimura

**Affiliations:** 1 Department of Ophthalmology and Visual Sciences, Kyoto University Graduate School of Medicine, Kyoto, Japan; 2 Department of Ophthalmology, Otsu Red Cross Hospital, Otsu, Japan

## Abstract

**Aim:**

To investigate the effect of inducible nitric oxide synthases (iNOS) on inflammatory reactions during endotoxin-induced uveitis (EIU) in mice by studying leukocyte–endothelial cell interactions.

**Methods:**

EIU was produced in immunosuppressed iNOS^−/−^ mice and C57BL/6 (normal) mice by footpad injection of lipopolysaccharide. Leukocytes were labelled with acridine orange. Leukocyte rolling in the retinal microcirculation was evaluated in vivo with acridine orange digital fluorography. The number of migrated leukocytes was counted in flat-mounted retina.

**Results:**

Both leukocyte rolling and migration peaked at 48 h after lipopolysaccharide injection. The maximal numbers of rolling leukocytes in the immunosuppressed iNOS^−/−^ mouse retina decreased by 98.2% (p<0.001) compared with that in the normal mouse retina at 48 h after lipopolysaccharide injection. In addition, the maximal numbers of migrated leukocytes in the immunosuppressed iNOS^−/−^ mouse retina decreased by 74.0% (p<0.001) compared with that in the normal mouse retina at 24 h after lipopolysaccharide injection. Furthermore, the diameters of major retinal veins of the immunosuppressed iNOS^−/−^ group were smaller at both 24 and 48 h after lipopolysaccharide injection than were those of the normal group (p<0.001, respectively).

**Conclusions:**

A lack of iNOS suppresses leukocyte–endothelial cell interactions in the retinas of mice with EIU. This suggests that iNOS may play a role in the management of patients with uveitis and other inflammatory conditions.

Leukocytes play a crucial role in inflammatory conditions by interacting with endothelial cells and migrating to the site of inflammation. In the inflamed area, endothelial cells are activated to express adhesion molecules that cause leukocyte–endothelial cell interactions through a multistep process.^1^ ^2^ Initially, leukocytes interact with P-selectin, which is expressed on endothelial cells, and begin rolling along vessel walls. The leukocytes then interact with intercellular adhesion molecule 1 (ICAM-1), adhere to endothelial cells and migrate out of the vessels. These leukocytes subsequently release cytokines and produce proteases and superoxide radical species, which also participate in the cascade of inflammation.^3^ ^4^ Because the reactions of these leukocytes cause inflammatory tissue injury or endothelial cell injury,^5^ ^6^ it is important to evaluate the behaviour of leukocytes in vivo.

Endotoxin-induced uveitis (EIU) is an ocular inflammation model created in an experimental animal by a subcutaneous injection of lipopolysaccharide,^7^ without direct exposure to the eye.^8^ ^9^ EIU is characterised by leukocyte infiltration with blood–ocular barrier disruption. In this model, inflammatory reactions have been reported to occur in both the anterior and posterior segments of the eye, including infiltration of leukocytes into the aqueous humour, vitreous cavity and retina.^8^^–^10

During EIU, the expression of inducible nitric oxide synthase (iNOS) is upregulated and is thought to play a key role in the pathogenesis of EIU.^11^ Nitric oxide (NO), derived from iNOS, regulates the expression of P-selectin and ICAM-1 and contributes to vasodilation.^12^^–^14 In addition, iNOS expression in the retina is likely to cause tissue damage by interfering with the beneficial activities of constitutive neuronal nitric oxide synthase (NOS) and endothelial NOS.^15^^–^17 Little is known, however, regarding the exact relationship between iNOS and tissue damage.

In-vivo methods to evaluate quantitatively leukocyte–endothelial cell interactions have been established in mouse retina.^18^ Using these methods, we can physiologically evaluate the number of rolling leukocytes and the number of migrated leukocytes in mouse retina. With the recent progress in gene technology, represented by various knockout or transgenic mouse strains, the expression of specific genes can now be easily modified. Accordingly, in-vivo observations of leukocyte rolling and migration in mouse retina now accurately evaluate the genetic regulation of leukocyte behaviour in the inflammatory response.

In the study described here, we quantitatively evaluated the influence of iNOS on leukocyte–endothelial cell interactions in the lipopolysaccharide-stimulated retina with the use of mice lacking iNOS.^19^

## METHODS

### Animal model

All experiments were performed in accordance with the ARVO Statement for the Use of Animals in Ophthalmic and Vision Research. Male pigmented C57BL/6J Jms Slc mice (normal group, 8–10 weeks old; n  =  84) were obtained from Japan SLC, Inc (Shizuoka, Japan); male immunosuppressed iNOS^−/−^ mice (iNOS^−^ group, 8–10 weeks old; n  =  84) were obtained from the Jackson Laboratory (Bar Harbor, Maine, USA). EIU was produced in mice by injecting 100 μg lipopolysaccharide (*Salmonella typhimurium*; Sigma Chemical Co, St Louis, Missouri, USA) diluted in 0.1 ml sterile saline into one hind footpad of each animal. Control mice received a footpad injection of saline alone. All mice were maintained in an air-conditioned room with a 12-h light/12-h dark cycle and given free access to water and food until they were used for the experiments.

### Leukocyte rolling in mouse retina

To evaluate leukocyte rolling during an episode of EIU in mouse retina, we used acridine orange (AO) digital fluorography, a method that has been used to evaluate leukocyte–endothelial cell interactions in mouse retina, as previously described.^18^ In brief, a scanning laser ophthalmoscope (Rodenstock Instruments, Munich, Germany), coupled with a computer-assisted image analysis system (Radius, San Jose, California, USA), made continuous high-resolution images of the fundus, which had been stained by metachromatic fluorochrome AO (Wako Pure Chemicals, Osaka, Japan), which emits a green fluorescence when it interacts with DNA. An argon blue laser was used as the illumination source, with a regular emission filter for fluorescein angiography, because the spectral properties of leukocytes stained with AO are similar to those stained with sodium fluorescein. For further analysis, the images obtained were recorded on an S-VHS videotape at the rate of 30 frames per second.

AO digital fluorography was performed at 4, 12, 24, 48, 72 and 96 h after lipopolysaccharide injection. Six different mice from each group were used at each timepoint.

Immediately before AO digital fluorography, the mice were anaesthetised with xylazine hydrochloride (4 mg/kg) and ketamine hydrochloride (10 mg/kg) and their pupils were dilated with 0.5% tropicamide and 2.5% phenylephrine hydrochloride. Body temperature was maintained at between 37°C and 39°C throughout the experiment. A contact lens was used to retain corneal clarity throughout the experiment. Each mouse had a catheter inserted into the femoral vein and was placed on a moveable platform. AO (0.01% solution in saline) was injected continuously through the catheter for 1 minute at a rate of 1 ml/minute. Rolling leukocytes were defined as leukocytes that moved at a velocity slower than that of free-flowing leukocytes. The number of rolling leukocytes was calculated from the number of cells per minute crossing a fixed area of the vessel at a distance two disc diameters from the optic disc centre. The flux of rolling leukocytes for each mouse was defined as the average of individual numbers of rolling leukocytes seen in all major veins.

The diameters of the major retinal vessels were measured at two disc diameters from the centre of the optic disc in monochromatic images recorded before AO injection. Each vessel diameter was calculated in pixels as the distance between the half-height points determined separately on each side of the density profile of the vessel image and converted into real values using the calibration factor. The averages of the individual arterial and venous diameters were used as the arterial and venous diameters for each mouse.

### Number of leukocytes migrating in mouse retina

The number of leukocytes migrating in mouse retina was evaluated in flat-mounted retina after AO digital fluorography. Six different mice from each group were used at each timepoint. Thirty minutes after the injection of AO, one eye from each of six mice was enucleated. Blood was collected to count the number of leukocytes in the peripheral blood with a haematology analyzer (ERMA, Tokyo, Japan). At the conclusion of the experiment, each mouse was killed with an overdose of anaesthesia.

The retina was carefully removed, and flat-mounts were prepared using fluorescence anti-fading medium (Vector Laboratories, Burlingame, California, USA). The retinas were then examined using fluorescence microscopy (FITC filter; Olympus Optical, Tokyo, Japan) and the numbers of fluorescent dots in the retina within four separate circles of 800 μm diameter next to the optic disc were counted. The average of the numbers within the four circles was considered the number of leukocytes migrated in the retina for each mouse.

### Statistical analysis

All values were expressed as mean (SEM). The data were analyzed by repeated-measures analysis of variance, with post hoc comparisons tested with the Fisher protected least significant difference procedure. Unpaired t tests were used to compare results between groups at matched follow-up timepoints after lipopolysaccharide injection. Differences were considered significant when probability values were less than 0.05.

## RESULTS

### Physiological data

Table 1 shows changes in physiological variables at various timepoints after lipopolysaccharide injection. There were no significant differences between the normal group and the iNOS^−^ group in peripheral leukocyte count, mean arterial blood pressure or heart rate.

**Table 1:** Physiological variables

	Control (SEM)	4 h (SEM)	12 h (SEM)	24 h (SEM)	48 h (SEM)	72 h (SEM)	96 h (SEM)
Normal							
WBC, ×10^3^/μl	8.6 (0.8)	7.8 (1.0)	7.3 (0.9)	7.7 (1.9)	13.3 (1.4)*	10.9 (1.1)	9.4 (1.1)
MABP, mm Hg	100 (5)	101 (4)	106 (5)	103 (7)	102 (4)	101 (6)	100 (6)
Heart rate, beats/min	287 (6)	287 (8)	282 (6)	285 (8)	287 (8)	282 (6)	283 (6)
iNOS knockout							
WBC, ×10^3^/μl	8.1 (0.3)	6.7 (1.3)	7.4 (0.8)	7.7 (0.5)	11.1 (0.5)*	9.6 (0.9)	8.7 (1.2)
MABP, mm Hg	98 (3)	96 (4)	97 (7)	98 (7)	100 (6)	98 (6)	99 (5)
Heart rate, beats/min	282 (5)	278 (7)	281 (5)	285 (7)	283 (7)	281 (6)	288 (6)

iNOS, inducible nitric oxide synthase; MABP, mean arterial blood pressure; WBC, white blood cell peripheral leukocyte count.

Values are means (SEM). n  =  6 at each timepoint in both groups.

There were no significant differences between the normal and iNOS knockout mice group.

*p<0.05 compared with control values in each group.

### Vessel diameters

**Figure 1:**
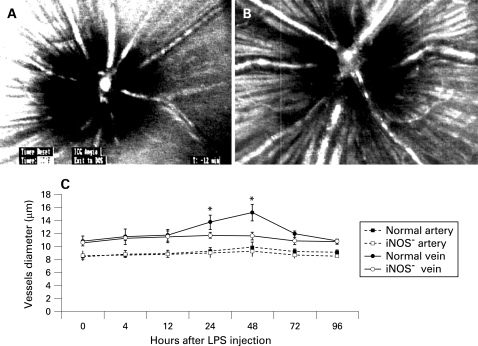
Digitised monochromatic images of major retinal vessels obtained with a scanning laser ophthalmoscope in a control mouse (A) and a mouse 48 h after lipopolysaccharide (LPS) injection (B). Time course of the diameters of major retinal arteries and veins after lipopolysaccharide injection (C). Values are mean (SEM). n  =  6 at each timepoint for each group. *p<0.05 compared with artery or vein of normal mice. iNOS, inducible nitric oxide synthase.

Fig 1A and B show characteristic fundus images of a control mouse and a mouse 48 h after lipopolysaccharide injection, respectively. Fig 1C indicates changes in major retinal vessel diameters in mice at various timepoints after lipopolysaccharide injection. The diameters of major retinal arteries and veins of the iNOS^−^ group were smaller than those of the normal group at all timepoints studied. In arteries of the iNOS^−^ group, slight vasoconstriction occurred and peaked at 48 h after lipopolysaccharide injection. At 48 h after lipopolysaccharide injection, the diameters of arteries in the iNOS^−^ group were reduced to 93.3% of those of the normal group (p = 0.340). In veins of the iNOS^−^ group, significant vasoconstriction occurred; this also peaked at 48 h after lipopolysaccharide injection, when the diameters of veins in the iNOS^−^ group were reduced to 76.4% compared with those of the normal group (p = 0.028).

### Leukocyte rolling

**Figure 2:**
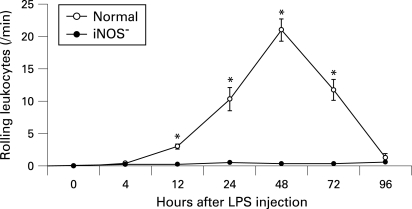
Time course of the flux of rolling leukocytes along major retinal veins after lipopolysaccharide (LPS) injection. Values are mean (SEM). n  =  6 at each timepoint for each group. *p<0.05 compared with normal mice. iNOS, inducible nitric oxide synthase.

Immediately after AO was infused intravenously, leukocytes were the only circulating blood cells that were stained, although vascular endothelial cells were stained faintly. In the non-treated control mice, no rolling leukocytes were observed. In the normal group, some leukocytes were observed to roll slowly along major retinal veins but not along any major retinal arteries. At 4 h after lipopolysaccharide injection, several leukocytes were observed rolling along the venous walls. The flux of rolling leukocytes then increased gradually and peaked at 48 h after lipopolysaccharide injection, but this flux of rolling leukocytes decreased to almost basal levels at 96 h after lipopolysaccharide injection. In the iNOS^−^ group, leukocyte rolling was significantly inhibited (fig 2, p<0.001). The maximal numbers of rolling leukocytes in the iNOS^−^ group were reduced by 98.2% (p<0.001) at 48 h after lipopolysaccharide injection compared with those in the normal group.

### Number of migrated leukocytes

**Figure 3:**
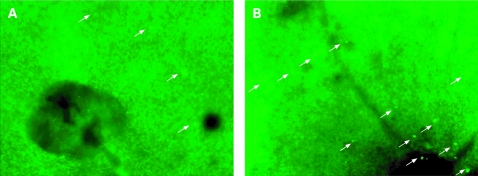
Migrated leukocytes in the retina were observed as fluorescent dots (arrows) at 30 minutes after acridine orange injection. A significant reduction in leukocyte migration was seen in the inducible nitric oxide synthase (iNOS) knockout mouse (A) compared with that of a normal mouse (B) at 48 h after lipopolysaccharide injection.

**Figure 4:**
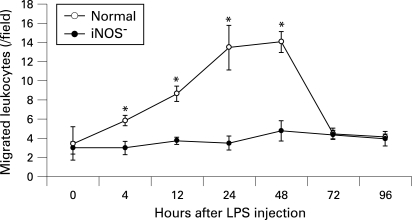
Time course of the number of leukocytes accumulating in the retinal microcirculation after lipopolysaccharide (LPS) injection. Values are mean (SEM). n  =  6 at each timepoint for each group. *p<0.05 compared with normal mice. iNOS, inducible nitric oxide synthase.

At 30 minutes after AO injection, leukocytes in the vessels, as well as endothelial cells, became faint because of the washout effect but migrated leukocytes in the retina could still be identified as distinct fluorescent dots with the highest contrast. Accordingly, we observed only those leukocytes that were exposed to a high concentration of AO in the vessels for a few minutes and that were extravasated before the washout effect. Fig 4 shows the numbers of leukocytes that migrated in the retina at each timepoint. Few leukocytes were found in control retina. At 4 h after lipopolysaccharide injection, the flux of migrated leukocytes began to increase and peaked at 48 h. In the iNOS^−^ group, leukocyte migration was significantly inhibited (p<0.001); the maximal numbers of migrated leukocytes in the iNOS^−^ group was reduced by 74.0% (p<0.001) at 24 h compared with migrated leukocytes in the normal mouse group (fig 3 and fig 4).

## DISCUSSION

In mouse retina with EIU, we demonstrated that a lack of iNOS attenuated leukocyte rolling along the major retinal veins and subsequent leukocyte migration into the retina. Moreover, in EIU mice, the diameters of major retinal arteries and veins of iNOS knockout mice were smaller than those of normal mice at all timepoints studied. On the basis of these findings, we suggest that iNOS might stimulate inflammation by leukocyte–endothelial cell interactions.

NO is a free radical produced in mammalian cells from l-arginine and oxygen by isoforms of an enzyme known as NOS: neuronal NOS, iNOS and endothelial NOS. The role of NO in inflammation is an extremely controversial area of research, with studies demonstrating both beneficial and deteterious effects after NOS inhibition.^20^ Mandai *et al*^11^ demonstrated that the expression of iNOS is upregulated during EIU and apparently plays an important role in the pathogenesis of EIU.^11^ The authors also noted that NO derived from iNOS regulates the expression of P-selectin and ICAM-1 and contributes to vasodilation. Also, Sennlaub *et al*^21^ reported that iNOS mediates retinal apoptosis in ischaemic proliferative retinopathy. In contrast, although the exact mechanisms are not fully understood, other groups have demonstrated that NO, released by either endothelial NOS or iNOS, modulates the leukocyte–endothelial cell interaction. Dal Secco *et al*^22^ reporterd that selective inhibitors of iNOS and endothelial NOS increase leukocyte rolling and adhesion to endothelial cells and increase neutrophil transmigration to sites of inflammation. In addition, Charlotte *et al*^23^ demonstrated that iNOS expressed in the outer retina might play a protective role in experimental autoimmune uveitis. In the current study, leukocyte rolling and migration in the immunosuppressed iNOS^−/−^ mouse retina were dramatically suppressed compared with the normal mouse retina after lipopolysaccharide injection. These dichotomous results might be because the biological effects of iNOS may be derived from various inflammatory processes at the level of the microcirculation.

Rosenbaum *et al*^24^ reported that leukocyte–endothelial dynamics were not the same in different vascular beds (iris, limbus, choroid) in the mouse eye and that the inflammatory response differed between these vascular beds. Our findings support their report in the aspect of the difference in status of microcirculation in the retina with EIU from that in normal retina and that in other vascular beds with EIU. Also, Becker *et al*^25^ demonstrated that deletion of the IL-8 receptor homologue reduced leukocyte migration but had minimal effects on leukocyte rolling with the use of iris rhodamine angiography. We think IL-8 might trigger leukocyte migration rather than rolling and iNOS might have suppressive effects on both leukocyte rolling and migration through regulation of P-selectin and ICAM-1.

In the present study, the diameters of major retinal arteries and veins of the iNOS^−^ group were smaller than those of the normal group at all times. In arteries of the iNOS^−^ group, slight vasoconstriction was seen after lipopolysaccharide injection, whereas the veins showed vasoconstriction compared with the normal group. In general, lipopolysaccharide-induced EIU causes significant vasodilation.^26^ It was previously demonstrated that diabetes reduced vein vasodilation in the retinas of rats with EIU, probably by the decreased expression of iNOS.^27^ This decreased expression of iNOS would also explain the suppressed leukocyte rolling and accumulation, because of its effect on the expression of P-selectin and ICAM-1.^12^^–^14 Our findings are consistent with these preceding reports.

In conclusion, we have demonstrated that a lack of iNOS attenuates leukocyte rolling along major retinal veins and the subsequent migration of leukocytes into the retinas of mice with EIU. To our knowledge, this study is the first to show that a lack of iNOS suppresses leukocyte–endothelial cell interactions in EIU of mouse retina. This suggests that iNOS may play a role in the management of patients with uveitis and other ocular inflammatory conditions.
